# Pubertal timing and adult fracture risk in men: A population-based cohort study

**DOI:** 10.1371/journal.pmed.1002986

**Published:** 2019-12-02

**Authors:** Liesbeth Vandenput, Jenny M. Kindblom, Maria Bygdell, Maria Nethander, Claes Ohlsson

**Affiliations:** 1 Centre for Bone and Arthritis Research, Department of Internal Medicine and Clinical Nutrition, Institute of Medicine, Sahlgrenska Academy, University of Gothenburg, Gothenburg, Sweden; 2 Bioinformatics Core Facility, Sahlgrenska Academy, University of Gothenburg, Gothenburg, Sweden; The Hospital for Sick Children, CANADA

## Abstract

**Background:**

Puberty is a critical period for bone mass accrual, and late puberty in boys is associated with reduced bone mass in adult men. The role of variations in pubertal timing within the normal range for adult fracture risk in men is, however, unknown. We, therefore, assessed the association between age at peak height velocity (PHV), an objective measure of pubertal timing, and fracture risk in adult men.

**Methods and findings:**

In the BMI Epidemiology Study Gothenburg, 31,971 Swedish men born between January 1, 1945, and December 31, 1961, with detailed growth data (height and weight) available from centrally archived school healthcare records and the conscription register were followed until December 31, 2016. Age at PHV was calculated according to a modified infancy–childhood–puberty model, and fracture information was retrieved from the Swedish National Patient Register. The mean ± SD age at PHV was 14.1 ± 1.1 years. In total, 5,872 men (18.4%) sustained at least 1 fracture after 20 years of age and 5,731 men (17.9%) sustained a non-vertebral fracture after 20 years of age during a mean ± SD follow-up of 37.3 ± 11.7 years. Cox proportional hazards models adjusted for birth year and country of origin revealed that age at PHV was associated with the risk of any fracture and non-vertebral fracture. Participants with age at PHV in the highest tertile (after 14.5 years of age) were at greater risk of any fracture (hazard ratio [HR] 1.15, 95% confidence interval [CI] 1.08–1.22, *P <* 0.001) and non-vertebral fracture (HR 1.16, 95% CI 1.09–1.24, *P <* 0.001) compared with those with age at PHV in the lowest tertile (at 13.6 years of age or younger). Additional adjustments for birthweight, childhood BMI, adult educational level, and young adult height did not attenuate the associations between age at PHV and adult fracture risk. Limitations of this study include the inability to adjust for important risk factors for fracture, inadequate power to assess the relation between pubertal timing and specific fracture types, and the limited generalizability to other populations.

**Conclusions:**

In this study, we observed that late pubertal timing was associated with increased adult fracture risk in men. These findings suggest that information on pubertal timing might aid in the identification of those men at greatest risk of fracture.

## Abstract 2

In a cohort study of men born in Sweden, Liesbeth Vandenput and colleagues investigate whether the timing of puberty can predict the risk of fracture later in life.

## Author summary

**Why was this study done?:**

- It is well known that pubertal timing is associated with peak bone mass and bone health later in life, with later puberty in boys being associated with reduced bone mass in adult men.
- However, it remains unknown whether pubertal timing is associated with the clinical endpoint fracture.
- This study investigates the predictive role of variations in pubertal timing for adult fracture risk in men.

**What did the researchers do and find?:**

- We conducted a population-based cohort study that included 31,971 Swedish men born in 1945–1961.
- Using age at peak height velocity as an objective measure of pubertal timing, we found that pubertal timing in boys was associated with adult fracture risk.
- Men with late pubertal timing (age at peak height velocity in the highest tertile) had a 15% higher risk of sustaining a fracture compared with those with early puberty (age at peak height velocity in the lowest tertile).

**What do these findings mean?:**

- These results highlight the importance of pubertal timing as a risk marker for adult fracture in men.
- These findings suggest that information on pubertal timing might help to identify those men at greatest risk of fracture.

## Introduction

Puberty, representing the transition from childhood to adulthood, plays an essential role in longitudinal and appositional bone growth as well as bone mineral acquisition [1]. The timing of pubertal maturation is highly variable, with the onset ranging from 8 to 12 and from 9 to 13 years of age in girls and boys, respectively [2–4]. Pubertal timing in girls is most often defined by the age at menarche, a distinct late marker of sexual development. In young adult women, later age at menarche is associated with impaired bone mass accrual, reduced mechanical strength, and increased fracture risk [5–9]. Retrospective epidemiological studies of pre- and postmenopausal women also demonstrated that later age at menarche was associated with lower areal and volumetric bone mineral density (BMD) [10–14] and increased risk of osteoporotic fractures [15–17]. In addition, recent Mendelian randomization analyses indicated that age at menarche might play a causal role in the etiology of osteoporosis since age at menarche was causally associated with BMD at both the lumbar spine and femoral neck in women [18,19]. In boys, age at voice breaking represents a distinct indicator of puberty, but its retrospective use has not been validated and may be limited due to recall bias [13]. Instead, age at peak height velocity (PHV), i.e., the age at which the maximum longitudinal growth velocity is attained, can be used as an objective measure of pubertal timing based on growth curve analysis [20]. Several observational studies demonstrated that older age at puberty in boys, assessed either by Tanner stage [6] or by age at PHV [9], inversely associated with areal bone density measurements in young adulthood. We previously showed that late age at PHV was associated with low trabecular and cortical volumetric BMD at the radius as well as previous fracture in 642 young adult Swedish men participating in the Gothenburg Osteoporosis and Obesity Determinants study [21]. Also, later puberty, based on growth tempo from serial height measurements, was associated with sustained lower trabecular volumetric BMD and reduced bone size and calculated bone strength at the radius, as well as lower areal BMD at the lumber spine and total hip, in 792 men at 60–64 years of age from a British birth cohort study [22]. Moreover, later recalled timing of voice breaking, indicative of later puberty, was nominally associated with a higher risk of self-reported osteoporosis, but the association did not remain significant after adjustment for multiple testing [13]. Most recently, using a Mendelian randomization approach, genetically determined later puberty was causally associated with lower areal BMD at the lumbar spine and femoral neck in adult men [18]. However, evidence supporting a role of pubertal timing in adult fracture risk in men is still missing, most likely because of lack of well-powered cohorts with long-term follow-up and an objective measure of male pubertal timing available. Therefore, the aim of this study was to investigate the predictive role of variations in pubertal timing within the normal range, assessed by age at PHV, for adult fracture risk in the large-scale population-based BMI Epidemiology Study (BEST) Gothenburg. As previous evidence indicated that pubertal timing in boys inversely associates with BMD in adult men, we hypothesized that late puberty may be a risk marker for adult fracture risk in men.

## Methods

This study is reported as per the Strengthening the Reporting of Observational Studies in Epidemiology (STROBE) guidelines (S1 Checklist).

### Study design and participants

**Fig 1:**
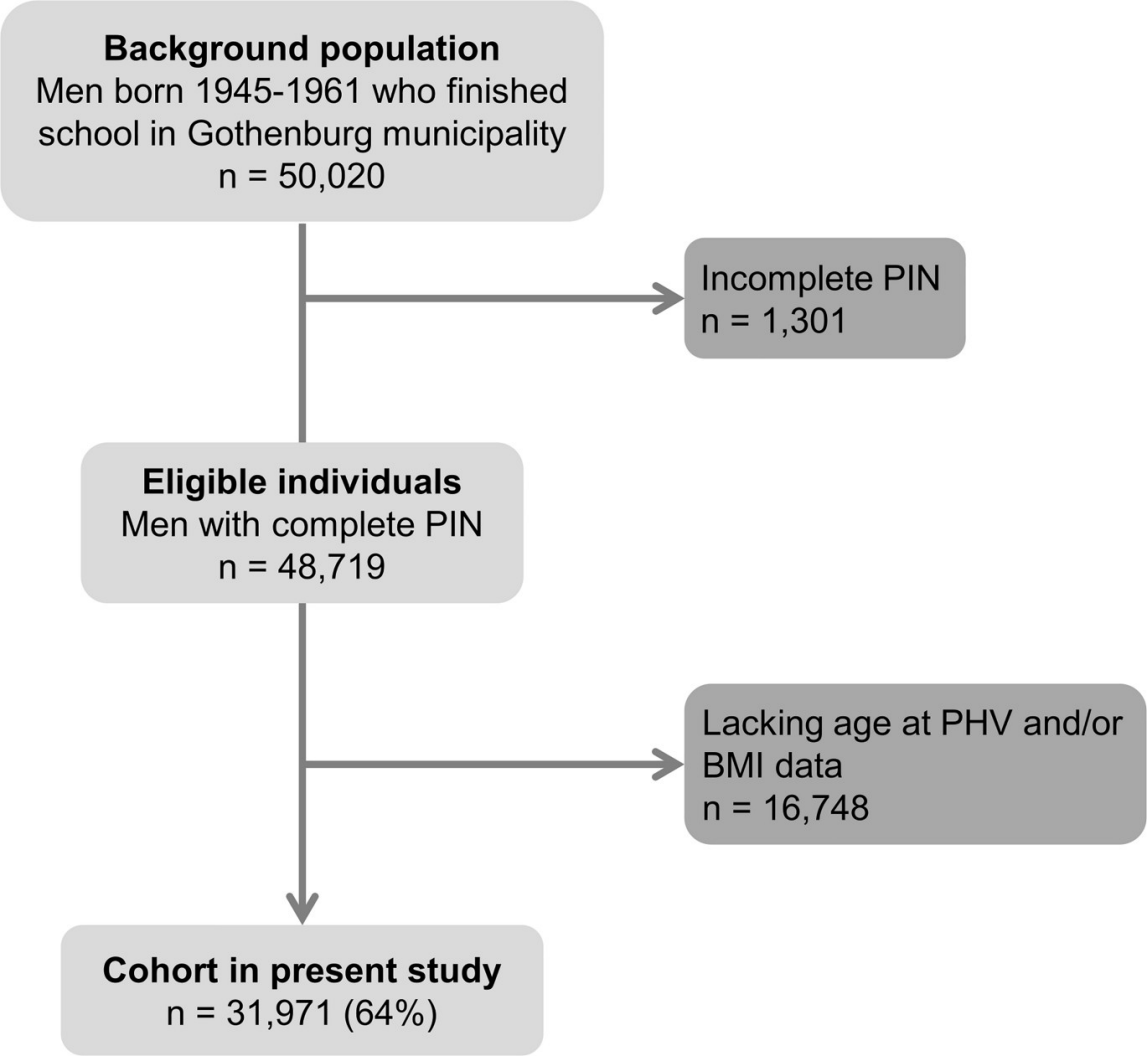
Flow chart of the BMI Epidemiology Study Gothenburg participants included in the present study. BMI, body mass index; PHV, peak height velocity; PIN, personal identity number.

The population-based BEST Gothenburg cohort was initiated with the overall aim to study the impact of childhood BMI and pubertal timing on diseases during adulthood [23–25]. To that end, we collected data on birthweight as well as directly measured height and weight from centrally archived school healthcare records for all men born between January 1, 1945, and December 31, 1961, who attended school in Gothenburg, Sweden, as previously described [23]. These measurements were performed by specially trained school nurses. For height measurements, a wall-mounted stadiometer was used, and the participants were weighed wearing lightweight clothing. These health examinations were performed according to a prespecified program throughout childhood and until the children finished secondary school. We also collected height and weight at young adult age from military conscription examinations. Conscription was mandatory until 2008 for all Swedish men. The study cohort was linked to high-quality national disease registers using the personal identity numbers (PINs) from the included individuals. Individuals eligible for the study were those with a school healthcare record in the central archive and a 10-digit PIN (Fig 1). For the present study, individuals with data available for calculation of BMI and age at PHV in the first BEST Gothenburg cohort [23] were included (*n =* 31,971; Fig 1 and Table 1). We compared BMI, height, and weight from the examination at conscription for those included in the present study (*n =* 29,305 individuals included in the study who also had a conscription exam record) with those not included (*n =* 5,386 individuals excluded based on missing age at PHV and BMI data but who had a conscription exam record). Minor differences regarding BMI (0.3% difference, mean ± SD, 21.14 ± 2.52 and 21.05 ± 2.57 kg/m^2^, respectively, *P* = 0.022 using *t* test), height (0.9% difference, 179.2 ± 6.4 and 178.7 ± 6.5 cm, respectively, *P* < 0.001), and weight (0.4% difference, 68.0 ± 9.4 versus 67.3 ± 9.6 kg, respectively, *P* < 0.001) were observed. Childhood BMI and age at PHV were a strong and predefined focus of the BEST Gothenburg cohort [23], but there was no detailed prospective statistical analysis plan for how to model the data with respect to adult fracture risk. The ethics committee at the University of Gothenburg approved the study and waived the requirement for oral and written informed consent.

**Table 1:** Characteristics of the study participants.

Characteristic	BEST Gothenburg cohort, born 1945–1961 (*n =* 31,971)
Childhood BMI (age 8 years, kg/m^2^)	15.7 (1.4)
Age at PHV (years)	14.1 (1.1)
Young adult height (cm)	179.3 (6.4)
Country of origin	
Sweden	26,749 (83.7%)
Other countries	5,222 (16.3%)
Participants with at least 1 incident fracture after 20 years of age	
All fractures	5,872 (18.4%)
Non-vertebral fractures	5,731 (17.9%)
Birthweight (kg)^a^	3.58 (0.55)
Adult educational level^b^	
Elementary school	5,548 (17.9%)
Secondary school	13,520 (43.6%)
University level	11,911 (38.4%)

Values are given as mean (SD) or number (percent).

^a^Birthweight was available in a subsample (*n =* 30,365).

^b^Adult educational level at 45 years of age was available in a subsample (*n =* 30,979).

BEST, BMI Epidemiology Study; BMI, body mass index; PHV, peak height velocity.

### Study exposures

To adequately calculate age at PHV in an unbiased manner, height measurements before, during, and after the pubertal period are required. We calculated age at PHV according to the infancy–childhood–puberty model [20], modified as previously described [21,26]. Overall, 13.3% of eligible participants were excluded since they did not have enough height measurements available to estimate a reliable age at PHV with our curve-fitting program. For each individual growth curve with sufficient information in all 3 growth phases, the infancy–childhood–puberty model was fitted by minimizing the sum of squares using a modification of the Levenberg–Marquardt algorithm [20]. Age at PHV was defined as the age at maximum growth velocity during puberty and was estimated by the curve-fitting program. PHV is generally believed to be reached within 2 years after pubertal onset [20,27]. Prepubertal childhood BMI at 8 years of age was calculated using all paired height and weight measurements in the period between 6.5 and 9.5 years of age and age-adjusted to 8 years of age using a linear regression model. Birthweight was retrieved from school healthcare records. Young adult height is the last recorded height from conscription examination or school healthcare records: this examination occurred after 17.5 years of age. Information on country of birth and migration was retrieved from the Longitudinal Integration Database for Health Insurance and Labour Market Studies held at Statistics Sweden. Country of origin was categorized as Sweden (the study participant and both his parents born in Sweden) or other countries (the participant or one or both parents not born in Sweden or information on country of birth missing). The study participants’ educational level at 45 years of age, categorized as elementary school, secondary school, or university level, was also retrieved from the Longitudinal Integration Database for Health Insurance and Labour Market Studies.

### Study outcomes

Dates and diagnoses for the first fracture event after 20 years of age were retrieved from the Swedish National Patient Register, initiated in 1964 and with full coverage in the Gothenburg region from 1972 onward. A diagnosis of a fracture was defined according to International Classification of Diseases (ICD) system codes (all fractures: S02 [except S02.5], S12, S22, S32, S42, S52, S62, S72, S82, and S92 in ICD-10 and 800–829 in ICD-8 and ICD-9; non-vertebral fractures: all of the above except S22.0, S22.1, and S32.0 in ICD-10, all of the above except 805C-F and 806C-F in ICD-9, and all of the above except 805.21, 805.31, 805.91, 806.21, 806.31, 806.91, 806.22, 806.32, and 806.92 in ICD-8). The 31,971 men included in the study were followed from 20 years of age until the first fracture event (any fracture, *n =* 5,872; non-vertebral fracture, *n =* 5,731), with censoring at migration (*n =* 2,372), death (*n =* 3,252), or study end (December 31, 2016), whichever came first. Individuals who were censored during the study (i.e., those who emigrated or died) were included in the study until the date of censoring.

### Statistical analysis

We used Cox proportional hazards regression to analyze the associations between age at PHV and fractures. The proportional hazards assumption was tested, and the Schoenfeld residuals plot for all fractures was visually assessed. No systematic deviations from proportionality were detected. Hazard ratios (HRs) and 95% confidence intervals (CIs) were estimated from the models and expressed per year increase in age at PHV in models in which age at PHV was entered as a continuous variable. All estimates were adjusted for birth year and country of origin. Additional adjustments were made for birthweight, childhood BMI at 8 years of age, adult educational level, and young adult height. During the peer review process, we also conducted analyses adjusted for young adult BMI and young adult weight. In predefined analyses, all fractures and non-vertebral fractures were analyzed. We lacked adequate statistical power to perform analyses according to fracture type. Possible nonlinearity in the association between age at PHV and fracture risk was evaluated by inclusion of a quadratic term in the Cox regression model. In response to peer review comments, we additionally used a restricted cubic spline approach in the Cox regression analysis for a flexible nonlinear assessment of the HR in relation to age at PHV. The number and positions of knots were selected using the Akaike information criterion (AIC). Four knots placed at the 5th, 33rd, 67th, and 95th percentile of age at PHV were found to give a small AIC and capture the average curve shape over a systematic assessment of different alternatives. Unadjusted Kaplan–Meier plots were used to illustrate fracture-free survival for study participants according to tertiles of age at PHV, and the log-rank test was used to assess the statistical significance of differences between the groups. In predefined sensitivity analyses, similar to those conducted previously [23,24], we examined a subpopulation including only participants with country of origin Sweden (*n =* 26,749) or excluding individuals (all fractures, *n =* 1,844; non-vertebral fractures, *n =* 1,828) with a first fracture event during the first 10 years of follow-up.

The parameters birthweight (*n =* 30,365, corresponding to 95% of the study cohort) and adult educational level (*n =* 30,979, corresponding to 97% of the study cohort) did not have a complete set of data. Models including birthweight and adult educational level included only the subgroup of men with birthweight and adult educational level available. Birthweight, childhood BMI, young adult weight, and young adult BMI were log-transformed and standardized when used in the Cox regression models. The restricted cubic spline analysis, the Kaplan–Meier survival plots, and the proportionality test were performed in R using the regression modeling strategies [28], survival [29], and survminer [30] packages; all other statistical analyses were performed in IBM SPSS Statistics (version 21.0; IBM, Armonk, NY, US).

## Results

### Study cohort

In this study, 31,971 Swedish men born between January 1, 1945, and December 31, 1961, with information on both BMI and age at PHV were included and followed until December 31, 2016 (Table 1; Fig 1). The mean ± SD age at PHV was 14.1 ± 1.1 years. The mean ± SD follow-up time starting from 20 years of age was 37.3 ± 11.7 years (1,190,921 person-years of follow-up) for all fractures and 37.3 ± 11.7 years (1,193,007 person-years of follow-up) for non-vertebral fractures. In total, 5,872 men (18.4%) sustained at least 1 fracture (rate of 4.9 per 1,000 person-years), and 5,731 men (17.9%) sustained a non-vertebral fracture (rate of 4.8 per 1,000 person-years) after 20 years of age.

### Pubertal timing is independently associated with fracture risk

**Fig 2:**
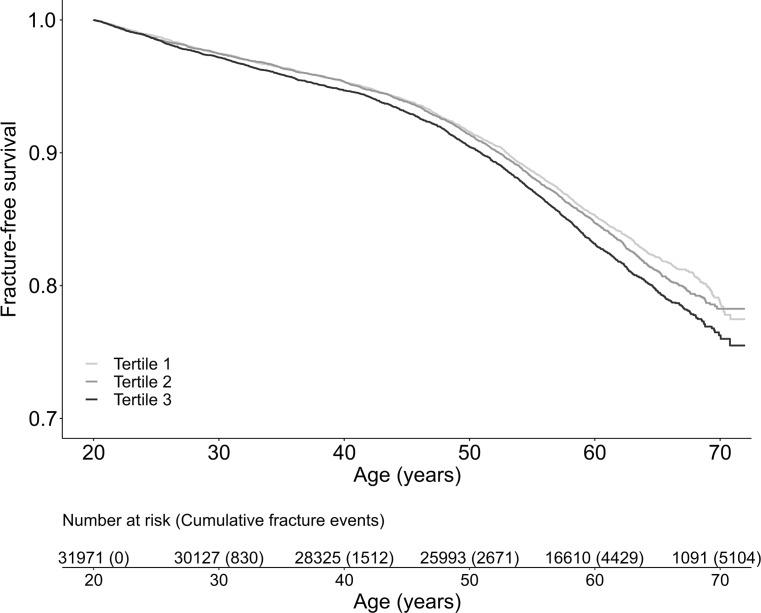
Kaplan–Meier curves of fracture-free survival according to pubertal timing. The graph shows fracture-free survival in 37,971 men followed for a mean of 37.3 years after 20 years of age according to whether participants had early (lowest tertile), average (middle tertile), or late (highest tertile) age at peak height velocity. The *P* value for comparison between groups assessed by the log-rank test was *P* = 0.186 for tertile 2 versus tertile 1 and *P* < 0.001 for tertile 3 versus tertile 1. Limits for age at PHV: tertile 1, ≤13.6 years of age; tertile 2, >13.6 and ≤14.5 years of age; tertile 3, >14.5 years of age.

Cox proportional hazards regression models adjusted for birth year and country of origin demonstrated that age at PHV was directly associated with the risk of any fracture (HR per year increase 1.05, 95% CI 1.03–1.08, *P* < 0.001) and non-vertebral fracture (HR per year increase 1.05, 95% CI 1.03–1.08, *P* < 0.001) when analyzed as a continuous variable. Quadratic models did not support a significant nonlinear association between age at PHV and any fracture (*P* = 0.099 for nonlinearity) or non-vertebral fracture (*P* = 0.114 for nonlinearity). Likewise, a restricted cubic spline analysis did not detect a significant nonlinear association between age at PHV and any fracture (*P* = 0.086 for nonlinearity) (S1 Fig). However, based on the appearance of the curve, participants with age at PHV above the 67th percentile, i.e., 14.5 years of age, appear to have an increased fracture risk. Additional exploratory analyses showed a significant association between age at PHV and adult fracture risk when the pubertal timing variable was entered as tertiles (early, average, and late age at PHV) in models adjusted for birth year and country of origin (all fractures, HR per tertile increase 1.07, 95% CI 1.04–1.11, *P* for trend < 0.001; non-vertebral fractures, HR per tertile increase 1.08, 95% CI 1.04–1.11, *P* for trend < 0.001). Men with age at PHV in the highest tertile (after 14.5 years of age) were at greater risk of any fracture (HR 1.15, 95% CI 1.08–1.22, *P* < 0.001) and non-vertebral fracture (HR 1.16, 95% CI 1.09–1.24, *P* < 0.001) compared with those with age at PHV in the lowest tertile (13.6 years of age or younger) (Table 2). Furthermore, participants with age at PHV in the highest tertile had an increased risk of any fracture (HR 1.10, 95% CI 1.03–1.17, *P* = 0.003) and non-vertebral fracture (HR 1.11, 95% CI 1.04–1.18, *P* = 0.001) compared with participants with age at PHV in the middle tertile. To further illustrate the association between age at PHV and adult fracture risk, we plotted Kaplan–Meier curves of fracture-free survival according to tertiles of age at PHV. These plots revealed an increased risk of any fracture for participants with age at PHV in the highest tertile compared with those with age at PHV in the lowest tertile (log-rank *P* < 0.001; Fig 2).

**Table 2:** Risk of adult fractures according to pubertal timing in men.

Outcome and age at PHV tertile	Base model		Model 1		Model 2	
	HR (95% CI)	*P* value	HR (95% CI)	*P* value	HR (95% CI)	*P* value
All fractures						
Tertile 1	Reference		Reference		Reference	
Tertile 2	1.05 (0.98–1.11)	0.168	1.03 (0.97–1.10)	0.337	1.03 (0.96–1.10)	0.367
Tertile 3	1.15 (1.08–1.22)	<0.001	1.15 (1.08–1.23)	<0.001	1.15 (1.08–1.23)	<0.001
Non-vertebral fractures						
Tertile 1	Reference		Reference		Reference	
Tertile 2	1.05 (0.98–1.11)	0.182	1.03 (0.96–1.10)	0.378	1.03 (0.96–1.10)	0.407
Tertile 3	1.16 (1.09–1.24)	<0.001	1.16 (1.09–1.25)	<0.001	1.17 (1.09–1.25)	<0.001

Cox proportional hazards regression models for adult fracture risk according to tertiles of age at PHV in 31,971 men followed for a mean of 37.3 years for both all fractures and non-vertebral fractures after 20 years of age. The base model is adjusted for birth year and country of origin (all fractures, *n =* 5,872; non-vertebral fractures, *n =* 5,731). Model 1 is further adjusted for birthweight, childhood BMI at 8 years of age, and adult educational level. Model 2 includes the covariates of model 1 as well as young adult height. Model 1 and model 2: *n =* 29,447; all fractures, *n =* 5,500; non-vertebral fractures, *n =* 5,368. Limits for age at PHV: tertile 1, ≤13.6 years of age; tertile 2, >13.6 and ≤14.5 years of age; tertile 3, >14.5 years of age.

PHV, peak height velocity.

Further adjustment of our regression models for birthweight, childhood BMI at 8 years of age, and adult educational level did not materially change the associations between age at PHV and the risk of any fracture or non-vertebral fracture (Table 2). Similarly, additional adjustment for young adult height did not attenuate the observed associations (Table 2). Finally, to address further potential confounding, the base model (adjusted for birth year and country of origin) was adjusted for young adult BMI and young adult weight. These adjustments did not alter the associations between age at PHV and the risk of any fracture or non-vertebral fracture (S1 Table).

### Sensitivity analyses

The observed associations between age at PHV and adult fracture risk were similar in a subpopulation including only participants with country of origin Sweden (*n =* 26,749; Table 3). In addition, in order to account for subclinical and/or undiagnosed diseases that could affect fracture risk, we performed analyses excluding the first 10 years of follow-up. This population restriction did not alter the associations between age at PHV and adult fracture risk (highest tertile versus lowest tertile: all fractures, HR 1.15, 95% CI 1.07–1.23, *P* < 0.001; non-vertebral fractures, HR 1.16, 95% CI 1.08–1.24, *P* < 0.001).

**Table 3:** Risk of adult fractures according to pubertal timing in men with country of origin Sweden.

Outcome and age at PHV tertile	HR (95% CI)	*P* value
All fractures		
Tertile 1	Reference	
Tertile 2	1.06 (0.99–1.13)	0.119
Tertile 3	1.15 (1.08–1.24)	<0.001
Non-vertebral fractures		
Tertile 1	Reference	
Tertile 2	1.06 (0.98–1.13)	0.128
Tertile 3	1.16 (1.09–1.25)	<0.001

Cox proportional hazards regression models for adult fracture risk according to tertiles of age at PHV in 26,749 men with country of origin Sweden followed for a mean of 37.7 years for all fractures and 37.8 years for non-vertebral fractures after 20 years of age. The models are adjusted for birth year (all fractures, *n =* 4,919; non-vertebral fractures, *n =* 4,803). Limits for age at PHV: tertile 1, ≤13.6 years of age; tertile 2, >13.6 and ≤14.5 years of age; tertile 3, >14.5 years of age.

PHV, peak height velocity.

## Discussion

The pubertal period has a key role in bone development. Although previous studies have shown that the timing of puberty in boys is associated with bone health later in life, it remains unknown whether the reduced bone mass in those with late compared with early puberty may be clinically important and consequently affect adult fracture risk in men. In this large-scale population-based cohort study, we demonstrate that late pubertal timing in boys, based on assessment of age at PHV, is a novel predictor of adult fracture risk. Specifically, men with late pubertal timing (age at PHV in the highest tertile) had a 15% higher risk of sustaining a fracture after 20 years of age compared with those with early puberty (age at PHV in the lowest tertile). This association remained significant after adjustment for confounding factors such as birthweight and prepubertal childhood BMI and was not attenuated after adjustment for young adult height.

We have previously demonstrated that late puberty, based on assessment of age at PHV, is associated with lower trabecular and cortical BMD and higher risk of prevalent fractures compared with early puberty in 19-year-old Swedish men participating in the Gothenburg Osteoporosis and Obesity Determinants study [21]. In the same cohort of young adult men, we have shown that these differences in bone health due to timing of puberty may be partially attenuated by catch-up growth, although some differences still remain, with deficits in areal BMD and volumetric BMD of the radius in men with late puberty [31]. Moreover, the effects of late puberty on bone health seem to partly persist into early old age as demonstrated by lower areal BMD at the lumbar spine and femoral neck as well as lower trabecular volumetric BMD and size and strength of the radius in 60- to 64-year-old men with late puberty [22]. Importantly, the present study is, to our knowledge, the first to evaluate the association between objectively assessed pubertal timing and adult fracture risk in men. In the BEST Gothenburg cohort, participants experiencing late puberty, i.e., PHV after 14.5 years of age, were at significantly greater risk of any fracture and non-vertebral fracture compared with those with early puberty, i.e., PHV at 13.6 years of age or younger. These results are in concordance with previous studies assessing the association between pubertal timing and fracture risk in women that demonstrated an increased adult fracture risk in women with a late age at menarche [15–17].

As we have previously shown that prepubertal childhood BMI at 8 years of age is inversely associated with age at PHV [26], we considered childhood BMI a potential confounder for our observed associations. However, adjustment for childhood BMI did not materially change the associations between pubertal timing and adult fracture risk. In addition, the observed associations were independent of adult educational level. Furthermore, the associations between pubertal timing and the risk of fractures were similar in sensitivity analyses excluding the first 10 years of follow-up. Finally, similar results were obtained in a subpopulation of men with country of origin Sweden as in the entire cohort, excluding a putative impact of changes in population structure on the observed associations.

Since this is an observational study, we can only hypothesize about the possible factors underlying the observed association between late pubertal timing and increased fracture risk. Previous studies by us and others have shown that late puberty in boys is associated with lower areal and volumetric BMD during adolescence and that this partly persists into early adulthood and early old age [21,22,31]. Also, Mendelian randomization analyses have recently suggested that boys who are genetically predisposed to later-than-average puberty, as indicated by later age at voice breaking, are more likely to have lower adult bone density [18]. Thus, BMD could potentially mediate the observed associations between late pubertal timing and increased fracture risk after 20 years of age in our study, especially given the well-recognized inverse association between BMD and fracture risk [32]. Unfortunately, BMD measurements were not available in our study to test the role of BMD in mediating the association between age at PHV and fracture risk. Later pubertal timing in boys is associated with increased adult height [31], which in turn is a recognized risk factor for fractures [33,34]. However, addition of young adult height to our Cox regression models did not attenuate the association between late puberty and increased risk of any fracture or non-vertebral fracture. This argues against a role for adult height in the association between late pubertal timing and increased adult fracture risk. Finally, one might speculate that late-maturing boys have lower sex steroid levels during puberty and this may persist into adulthood. Lower levels of androgens and estrogens later in life could consequently predispose individuals for osteoporosis and increased fracture risk in old age, as we and others have shown that low sex steroid levels are associated with an increased risk of fracture in older men [35,36]. Unfortunately, serum sex steroid levels were not available in our study to test this hypothesis.

This study has a number of strengths. To our knowledge, the BEST Gothenburg cohort is the first large population-based cohort of men with an objective assessment of pubertal timing available over the entire pubertal period. The near-complete participation in the free school healthcare program with repeated standardized measurements of height and weight strongly reduces the potential for selection bias. Moreover, the high-quality Swedish national disease registers allow for an extended and complete adult follow-up. We also acknowledge some limitations. We could not control for several important risk factors for fractures such as smoking, parental history of fractures, physical activity, and falls. In addition, we lacked statistical power to investigate the relation between pubertal timing and specific fracture types. Moreover, the number of vertebral fractures may be underestimated in our study since the fracture diagnosis is retrieved from patient registers. Unfortunately, we were not able to address potential confounding by adiposity since no adiposity measures are available in our cohort, but instead adjusted our models for young adult BMI, a surrogate marker for adiposity, and young adult weight. These adjustments did not affect our main findings. Also, we adjusted for young adult height in our analyses but cannot fully exclude remaining confounding effects of height. Environmental factors such as nutrition, climate, and endocrine disruptors are known to influence the age of pubertal onset in girls and boys [4]. The effects of endocrine-disrupting chemicals are compound-specific and vary according to the window of exposure [37]. Boys with moderate exposure to the estrogenic-acting bisphenol A during peripuberty had earlier pubertal timing compared with boys with the least bisphenol A exposure [38]. Also, prenatal exposure to flame retardants with estrogenic and androgenic properties was associated with earlier pubertal timing in boys [39]. On the other hand, current exposure to phthalates, which have antiandrogenic and obesogenic properties, was not associated with pubertal onset in boys [40]. Such data are, however, not available in our study. The present cohort primarily includes men of European origin, and therefore, the results may have limited generalizability to other ethnicities. Finally, the participants were born between 1945 and 1961; therefore, our findings may not apply to later-born cohorts, taking into account the potential confounding effect of a secular decline in pubertal timing in boys [41].

Osteoporosis and associated fragility fractures are an important public health concern due to a vastly increasing elderly population. It is, therefore, important to be able to predict which individuals are at elevated risk of fracture. In clinical practice, this is achieved by combining bone density imaging and clinical risk factors for fracture, often incorporated in fracture risk prediction tools [42]. Our findings suggest that late pubertal timing is a novel, independent risk factor for adult fracture risk in men that could support the identification of those men at highest risk of fracture.

In conclusion, in this population-based cohort study based in Sweden, we find an association between late pubertal timing and increased adult fracture risk in men. These findings suggest that information on pubertal timing might aid in the identification of those men at greatest risk of fracture.

## Supporting information

S1 Checklist STROBE checklist. (DOCX)

S1 Fig Smoothed plot of the likelihood of any fracture according to pubertal timing in men. (TIFF)

S1 Table Risk of adult fractures according to pubertal timing in men, adjusted for young adult BMI and young adult weight. (DOCX)

## Abbreviations

BEST: BMI Epidemiology Study
BMD: bone mineral density
CI: confidence interval
HR: hazard ratio
PHV: peak height velocity
PIN: personal identity number
