# Supplementary figures and images for: Pubertal timing and adult fracture risk in men: A population-based cohort study
Source: PLoS Med. 2019 Dec 2;16(12):e1002986. doi: 10.1371/journal.pmed.1002986 (PMC6886748; doi:10.1371/journal.pmed.1002986)

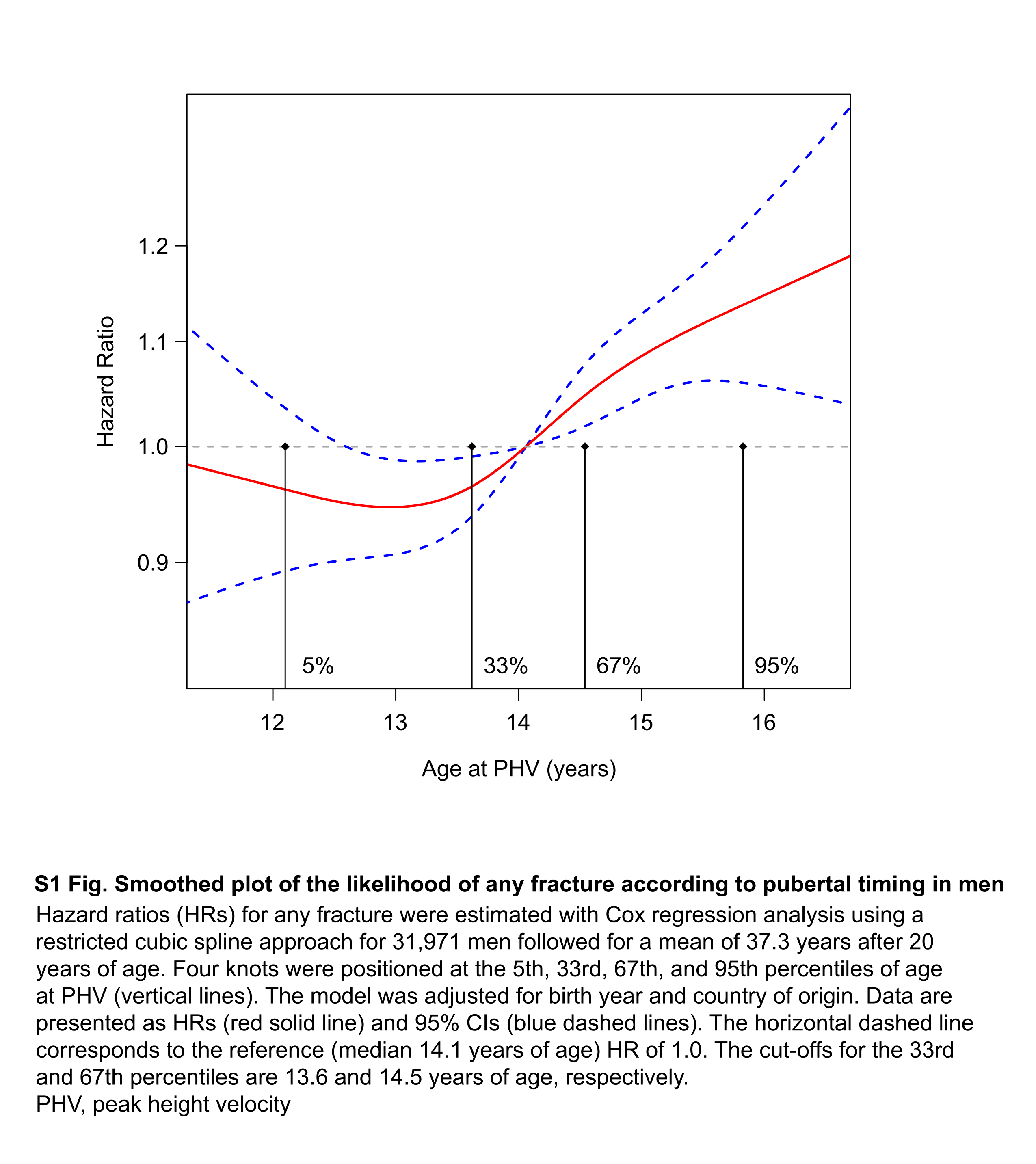

Supplement: S1 Fig — (TIFF) [file pmed.1002986.s002.tiff]
