# Supplementary material for: Pubertal timing and adult fracture risk in men: A population-based cohort study
Source: PLoS Med. 2019 Dec 2;16(12):e1002986. doi: 10.1371/journal.pmed.1002986 (PMC6886748; doi:10.1371/journal.pmed.1002986)
Supplement: S1 Table — (DOCX) [file pmed.1002986.s003.docx]

**S1 Table. Risk of adult fractures according to pubertal timing in men adjusted for young adult BMI and young adult weight.**

| **Outcome and age at PHV tertile** | **Base model** | | **Base model + young adult BMI** | | **Base model + young adult weight** | |
| --- | --- | --- | --- | --- | --- | --- |
|  | **HR (95% CI)** | ***P* value** | **HR (95% CI)** | ***P* value** | **HR (95% CI)** | ***P* value** |
| All fractures |  |  |  |  |  |  |
| Tertile 1 | Reference |  | Reference |  | Reference |  |
| Tertile 2 | 1.05 (0.98-1.11) | 0.168 | 1.05 (0.98-1.12) | 0.168 | 1.05 (0.98-1.12) | 0.165 |
| Tertile 3 | 1.15 (1.08-1.22) | <0.001 | 1.15 (1.08-1.22) | <0.001 | 1.14 (1.07-1.22) | <0.001 |
| Non-vertebral fractures |  |  |  |  |  |  |
| Tertile 1 | Reference |  | Reference |  | Reference |  |
| Tertile 2 | 1.05 (0.98-1.11) | 0.182 | 1.05 (0.98-1.12) | 0.183 | 1.05 (0.98-1.12) | 0.183 |
| Tertile 3 | 1.16 (1.09-1.24) | <0.001 | 1.16 (1.09-1.24) | <0.001 | 1.15 (1.08-1.23) | <0.001 |

Cox proportional hazards regression models for adult fracture risk according to tertiles of age at PHV in 31 971 men followed for a mean of 37.3 years for both all fractures and non-vertebral fractures after 20 years of age. The base model is adjusted for birth year and country of origin (all fractures, n=5,872; non-vertebral fractures, n=5,731) with additional adjustments for young adult BMI and young adult weight.

Limits for age at PHV: tertile 1, ≤ 13.6 years of age; tertile 2, > 13.6 and ≤ 14.5 years of age; tertile 3, > 14.5 years of age.

PHV, peak height velocity.
